# Perfluorocarbon liquid as a short-term tamponade for managing severe open globe injuries

**DOI:** 10.1186/s40942-025-00659-4

**Published:** 2025-03-25

**Authors:** Liang Li, Xinghong Sun, Mengru Su, Xiaofang Wang, Feng Jiang

**Affiliations:** Department of Ophthalmology, Nanjing Drum Tower Hospital, Affiliated Hospital of Medical School, Nanjing University, No.321 Zhongshan Rd, Nanjing, 210008 Jiangsu China

**Keywords:** Open globe injuries, Perfluorocarbon liquid tamponade, Staged vitrectomy, Retinal detachment, Traumatic choroidal rupture

## Abstract

**Purpose:**

To explore the use of perfluorocarbon liquids (PFCLs) as a short-term tamponade in a staged vitrectomy approach for managing severe open globe injuries (OGIs).

**Methods:**

This retrospective, interventional case series included patients undergoing 23-gauge pars plana vitrectomy with PFCL tamponade for 7–14 days (mean 11.2 ± 3.36 days), followed by secondary vitrectomy and silicone oil exchange. Key outcome measures included retinal and choroidal reattachment rates, best-corrected visual acuity (BCVA), intraocular pressure (IOP), and postoperative complications. Statistical analyses were performed using McNemar’s test and Student’s t-test.

**Results:**

Five eyes from five patients (mean age 55.6 ± 10.12 years; male to female ratio 4:1) were included, with a mean follow-up of 8.4 ± 4.9 months. All patients sustained zone II and III globe ruptures secondary to blunt trauma, with baseline visual acuity ranging from light perception (LP) to no light perception (NLP). Intraoperative findings included total hyphema, funnel retinal detachment, traumatic choroidal rupture (TCR), suprachoroidal hemorrhage (SCH) and extensive intraocular hemorrhage (EIH). Following PFCL removal, SCH exhibited full or partial resolution, and the posterior retina remained attached in all cases. All eyes were salvaged, and visual acuity improved to hand motion (HM) on postoperative day 1, maintaining stability throughout follow-up (P = 0.03682). No significant IOP changes were observed postoperatively (preoperative: 9.66 ± 2.38 mmHg; postoperative: 9.48 ± 3.31 mmHg, P = 0.9063). Retinal and choroidal attachment were maintained during follow-up, with no cases of phthisis bulbi, endophthalmitis, recurrent hyphema, or ocular hypertension. One patient developed corneal degeneration three months postoperatively.

**Conclusions:**

Short-term PFCL tamponade in a staged vitrectomy may facilitates retinal and choroidal stabilization while minimizing complications, offering a viable alternative for managing severe OGIs.

**Supplementary Information:**

The online version contains supplementary material available at 10.1186/s40942-025-00659-4.

## Introduction

Open globe injuries (OGIs) are a major cause of severe visual impairment, particularly in working-age individuals, with a higher prevalence among males in occupational settings [1, 2]. Despite the generally poor visual prognosis following OGIs, the application of pars plana vitrectomy (PPV) after initial globe repair has significantly enhanced the potential for globe preservation and improved visual outcomes [3]. Managing severe OGIs presents significant challenges due to the high incidence of posterior segment complications, such as choroidal ruptures, extensive SCH, and intraocular hemorrhage. One of the most critical concerns is the risk of ocular venous air embolism (OVAE), a potentially fatal complication that occurs when pressurized air inadvertently enters the systemic circulation through torn vortex veins during fluid-air exchange (FAX) [4, 5].

Given these risks and the limitations of conventional surgical approaches, perfluorocarbon liquids (PFCLs) have emerged as a promising short-term tamponade due to their high density, optical clarity, and chemical inertness [6]. The intraoperative use of PFCLs in traumatic retinal detachment has been well-documented, as they effectively immobilize the detached retina and facilitate subretinal fluid drainage through peripheral breaks [7–9]. In this study, we aim to employ PFCLs as a short-term intraocular tamponade to stabilize the retina and choroid structure, leveraging their unique properties, including incompatibility with blood and a higher contact angle compared to silicone oil and gas [10].This study seeks to establish a safe and effective surgical approach for addressing challenging OGI cases involving complex retinal and choroidal injuries.

## Methods

### Patients

This retrospective case series included patients with severe OGIs who underwent short-term PFCL tamponade between July 2023 and December 2024.

### Surgical technique

Primary vitrectomy was performed within one week of initial scleral repair using a 23-gauge vitrectomy system (Constellation; Alcon, Fort Worth, TX, USA) with a Zeiss Resight 700 viewing system. Recombinant tissue plasminogen activator (rTPA) (50 μg) was injected intravitreally 4–24 h prior to vitrectomy to facilitate blood liquefaction. First, three paracenteses were made at the limbus, one for anterior chamber infusion, and the other two were used to remove hemorrhage and fibrotic tissue in the anterior chamber. Any opacified or dislocated lens material was removed to restore a clear optical pathway. The vitreous base was meticulously cleared to expand the surgical field, so that the vitrectomy incisions could be set at the pars plana. Retinal and choroidal damage was carefully assessed. SCH was externally drained through a scleral incision through the pars plana area, and the liquefied blood was then drained to facilitate choroidal apposition, with any remaining SCH left for self-absorption. Chorioretinectomy was performed for tissue incarceration release, and choroidal suturing was conducted for structural repositioning. Laser photocoagulation was performed as needed. Intraocular endoscopy was employed for severe corneal opacities. PFCL (F-Octane, FLUORON Gmbh) used to stabilize the posterior segment for 7–14 days, followed by secondary vitrectomy with silicone oil tamponade. After PFCL tamponade, patients were instructed to maintain a supine position as much as possible. A demonstration of the surgical procedure is provided in the supplementary video clip.

### Statistical analysis

SPSS 15.0 (SPSS Inc., Chicago, IL, USA) was used for data analyses. BCVA was assessed using a Snellen chart and IOP was measured via Goldmann applanation tonometry. A paired t-test was used for comparison of IOP between preoperative baseline and 1 month after surgery. Visual acuity results were categorized as NLP/LP and HM or better and McNemar's Chi-Square Test was used to analyze VA differences before and after surgery. A two-tailed value of P < 0.05 was considered statistically significant.

## Results

Five eyes from five patients (mean age: 55.6 ± 10.12 years) were analyzed, all presenting with globe ruptures (zone II/III) following blunt trauma. Mean follow-up was 8.4 ± 4.9 months. The mean follow-up time was 8.4 ± 4.9 months. The demographic characteristics and clinical outcomes of the patients are summarized in Table 1.

**Table 1:** Demographic characteristics and clinical outcomes of patients with severe open globe injuries

Case	Age/Gender		Initial VA	Pre & Intraoperative Evaluations	Type of Injury	Zone*	OTS	PFCL (days)	Final VA	Post-op IOP (mmHg) Mean ± SD	Follow-up (Months)
1	54/M		NLP	270° iris tear, traumatic cataract, funnel RD with incarceration, TCR, EIH	Rupture	III	1	7	HM	11.1 ± 2.36	18
2		68/F	LP	Total hyphema, funnel RD with incarceration, EIH, traumatic aniridia and aphakia	Rupture	III	1	14	HM	7.67 ± 2.13	7
3		67/M	NLP	Total hyphema, funnel RD, TCR, SCH, EIH, traumatic aniridia and aphakia	Rupture	II	1	7	HM	5.875 ± 0.922	8
4		45/M	NLP	Corneal laceration, total hyphema, funnel RD with incarceration, TCR, SCH, EIH, traumatic aniridia and aphakia	Rupture	III	1	14	HM	14 ± 1.414	5
5		44/M	LP	Corneal laceration, total hyphema, SCH, funnel RD with incarceration, TCR, EIH, traumatic aniridia and aphakia, ciliary body detachment	Rupture	III	1	14	HM	7.425 ± 1.764	4

VA, visual acuity, RD, retinal detachment; TCR, traumatic choroidal rupture; NLP, no light perception, LP, light perception, HM, hand motion; SCH, suprachoroidal hemorrhage; EIH, Extensive intraocular hemorrhage; IOP, intraocular pressure; OTS, ocular trauma score

*Zone II, full-thickness wound involves the sclera no more posteriorly than 5 mm from the corneoscleral limbus; Zone III: full-thickness wound posterior to zone II.

Slit-lamp examination revealed anterior chamber disorganization, corneal edema and traumatic iris root disinsertion. Total hyphema, traumatic aniridia and aphakia were present in four cases. B-scan ultrasonography indicated vitreous hemorrhage with suspected retinal and choroidal detachment. Ocular Trauma Score (OTS) classified all patients as OTS 1, indicative of poor prognosis [11]. Intraoperative assessments identified funnel retinal detachment and extensive intraocular hemorrhage in all cases. Retinal incarceration and SCH were presented in four cases. Following PFCL tamponade (mean duration: 11.2 days), SCH was partially or fully resorbed, and the posterior retina remained attached in all eyes. Visual acuity significantly improved to HM on postoperative day 1 (P = 0.03682) and remained stable throughout follow-up visits. IOP showed no significant change postoperatively (9.48 ± 3.31) compared to preoperative measurements (9.66 ± 2.38) (P = 0.9063) (Table 2). Retinal and choroidal attachment was maintained in all cases during the follow-up period. No cases of phthisis bulbi, endophthalmitis, recurrent hyphema or ocular hypertension were observed. One patient developed corneal degeneration three months postoperatively.

**Table 2:** Comparison of postoperative BCVA and IOP with preoperative data

	BCVA (n = 5)					IOP (mmHg)		
	NLP	LP	HM	χ^2^ Value	P	Mean ± SD	t Value	P
Preoperation	3	2	0	3.2	0.03682	9.66 ± 2.38	0.1253	0.9063
Postoperation	0	0	5			9.48 ± 3.31		

BCVA, Best-Corrected Visual Acuity; IOP, Intraocular Pressure

**Fig. 1:**
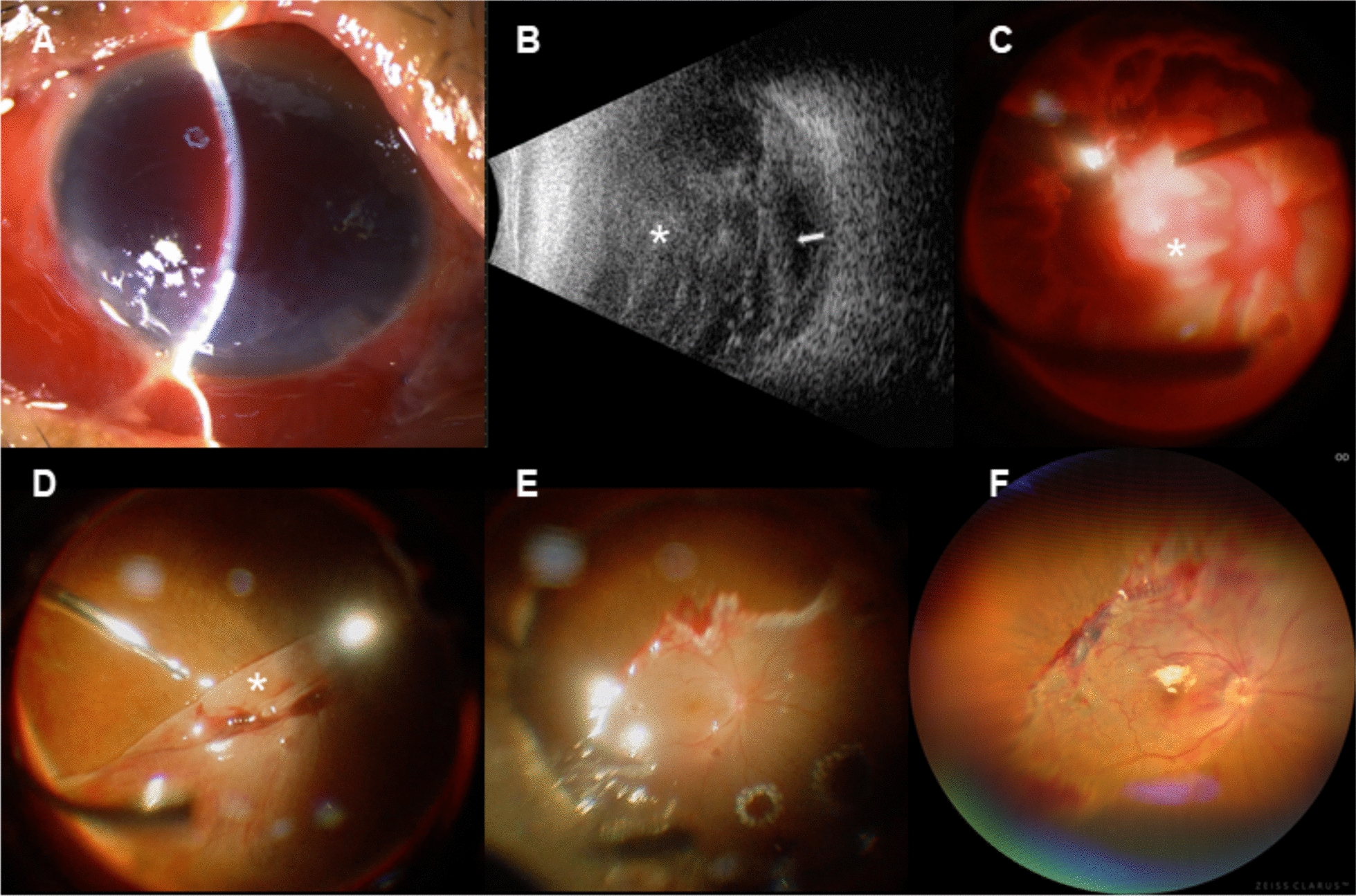
Case 2 presentation. **A** Slit-lamp examination showed corneal edema and total hyphema. **B** B-scan revealing massive vitreous hemorrhage (asterisk) and suspected retinal detachment (white arrow). **C** Primary vitrectomy demonstrating full retinal detachment (asterisk) with extensive lacerations and active bleeding. **D** Posterior retina remained attached under heavy water, with retinal folds present at the retinectomy border (asterisk) during secondary vitrectomy. **E** PFCL was refilled to unfold the peripheral retina, followed by radial retinotomy and endolaser retinopexy. **F** Color fundus photography showing an intact and attached posterior retina at the 1-month postoperative visit.

Case 2 A 68-year-old woman presented with a globe rupture in her right eye following a fall. On initial examination, her visual acuity was LP, and slit-lamp assessment revealed corneal edema with total hyphema (Fig. 1A). B-scan ultrasonography indicated massive vitreous hemorrhage with suspected retinal detachment (Fig. 1B). Five days after the initial scleral repair, she underwent primary vitrectomy. Intraoperatively, a complete retinal detachment with extensive lacerations and active intraocular bleeding was observed (Fig. 1C). A retinectomy was performed, and PFCL tamponade was applied to control bleeding and flatten the posterior retina. The patient was instructed to maintain a face-up position postoperatively for 14 days. During the secondary vitrectomy, the retina remained attached under PFCL, though retinal folds were present at the retinectomy border. (Fig. 1D). Radial retinotomy and endolaser photocoagulation were performed (Fig. 1E), followed by PFCL removal and silicone oil tamponade. The patient's vision improved to HM and IOP was 6.1 mmHg on postoperative day 1. The retina remained attached throughout follow-up (Fig. 1F).

**Fig. 2:**
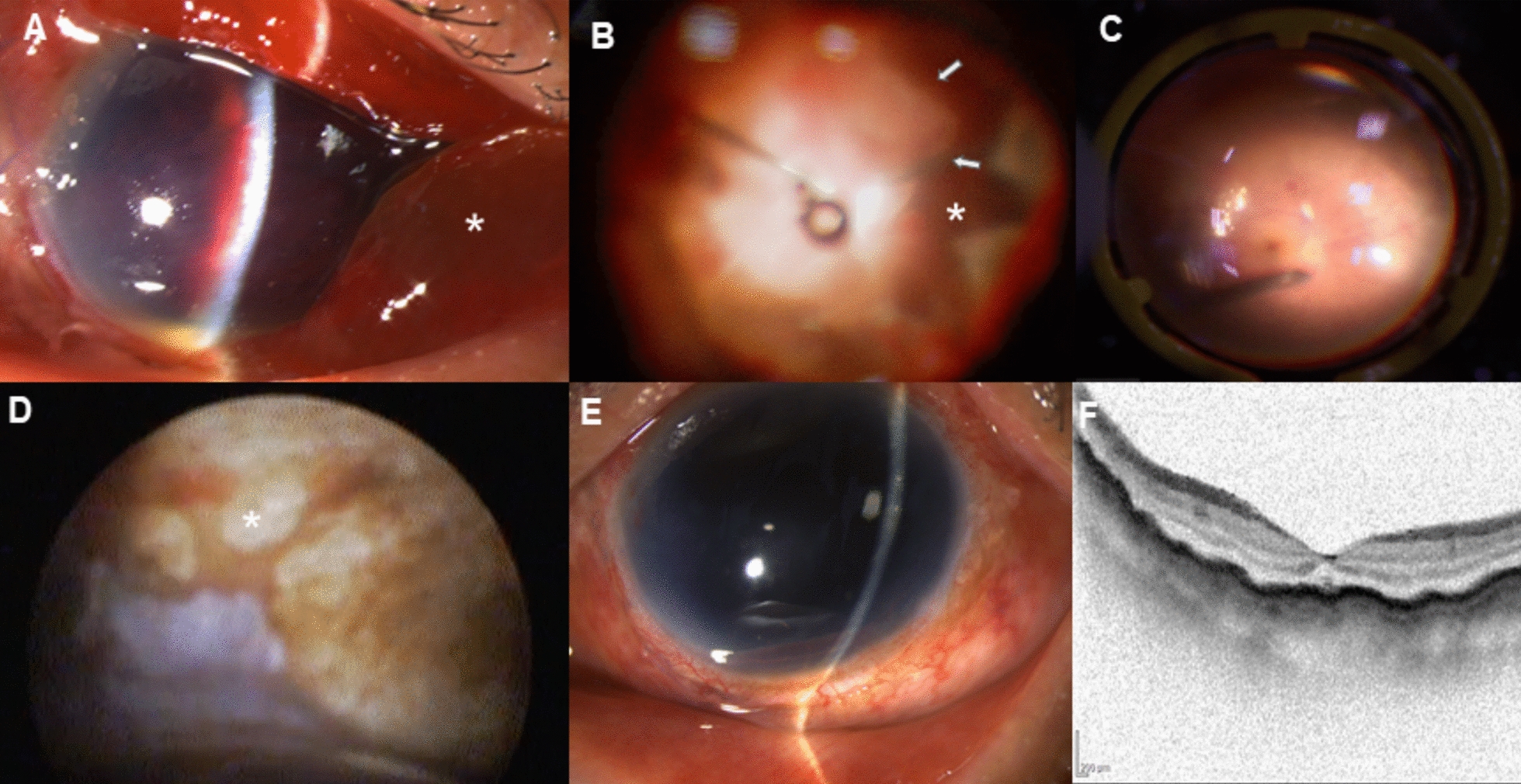
Case 3 presentation. **A** Slit-lamp examination showing total hyphema with extensive ocular tissue extrusion (asterisk). **B** Retinal margins after extensive retinectomy (white arrows) and highly detached choroid (asterisk) before PFCL injection. **C** Posterior retina remained attached after PFCL tamponade for 7 days. **D** Intraocular endoscopy revealing multiple choroidal defects in the peripheral region and scleral exposure (asterisk). **E** Slit-lamp examination showing mild corneal edema, aniridia, and aphakia with no inflammatory reaction. **F** OCT confirming attached macular retina 3 weeks postoperatively.

Case 3 A 67-year-old man presented with complete vision loss (NLP) after a blunt injury to his left eye from a polisher handle. Slit-lamp examination showed total hyphema with extensive ocular tissue extrusion (Fig. 2A). Primary vitrectomy was performed four days after scleral repair. Due to extensive vitreous hemorrhage, an intravitreal rTPA injection was administered preoperatively. Intraoperatively, a funnel retinal detachment with extensive vitreous hemorrhage, TCR and SCH was observed. Partial external suprachoroidal drainage and an extensive retinotomy were performed (Fig. 2B), followed by PFCL tamponade for seven days. During the secondary vitrectomy, intraocular endoscopy was required due to corneal opacity. The retina at the posterior area remained attached under PFCL (Fig. 2C). Intraocular endoscopy revealed multiple choroidal defects in the peripheral region and scleral exposure was noted (Fig. 2D). The patient’s visual acuity improved to HM on postoperative day 1. Follow-up exam showed mild corneal edema with no signs of inflammatory reaction (Fig. 2E). OCT imaging confirmed stable posterior retinal attachment (Fig. 2F).

## Discussion

The treatment of OGIs with NLP has long been debated, particularly regarding the potential for improving visual outcomes. Enucleation or evisceration was considered preferable for NLP eyes to reduce the risk of developing sympathetic ophthalmia (SO) [12–14]. However, recent studies have shown that the incidence of sympathetic ophthalmia after ocular trauma is very low, with reported incidence rate ranging from 0.12% to 0.19%. Moreover, no evidence has demonstrated that primary enucleation or evisceration significantly reduces the risk of secondary SO [15, 16]. Despite this, psychological distress, including depression and anxiety, is common following enucleation, particularly in acute settings where patients may not have had time to process the trauma [17]. Preserving the globe, therefore, offers advantages such as the potential for improved mobility with an ocular prosthesis and better preservation of the patient’s psychological well-being [18].

Advancements in vitrectomy techniques and instrumentation led to a shift in which more vitreoretinal surgeons recognizing the potential benefits of vitrectomy in treating severe OGIs. Given the heterogenous nature of ocular trauma, indexes such as OTS have been used to predict visual outcomes. Some studies suggest that poor visual acuity on presentation, retinal detachment and severe choroidal injuries are important prognostic factors for determining final visual acuity [19, 20]. However, other studies argue that vision recovery from NLP cannot be reliably predicted preoperatively, either by the OTS or by presenting clinical features [21–24]. Even in severe OGIs, vitrectomy has been shown to result in significant anatomical and functional improvements [25]. Feng et al. analyzed prognostic outcomes of OGI patients with NLP following vitrectomy, and result shown that 55% patients with NLP has gained visual acuity of LP or better after vitrectomy [26]. Therefore, it is recommended that all patients with OGIs undergo primary repair to allow for potential visual recovery [27].

Managing severe OGIs remains challenging due to extensive posterior segment damage, including retinal incarceration, choroidal rupture, choroidal avulsion, and SCH, which are often unpredictable preoperatively [19, 21]. Several risk factors contribute to unfavorable outcomes (phthisis bulbi or enucleation), such as globe rupture, zone III injuries, ciliary body damage, severe intraocular hemorrhage, closed funnel retinal detachment and choroidal damage [26]. In our cases, three out of five patients presented with NLP. Intraoperative evaluation revealed severe tissue damage in all patients, such as funnel retinal detachment, traumatic choroidal rupture, SCH and massive intraocular hemorrhage. These findings correspond to an OTS of 1, indicating the poorest prognosis according to Kuhn’s proposol [11]. Based on the severity and current prognosis studies [19, 26], these cases are likely to progress to phthisis bulbi and blindness, even with timely vitrectomy.

**Fig. 3:**
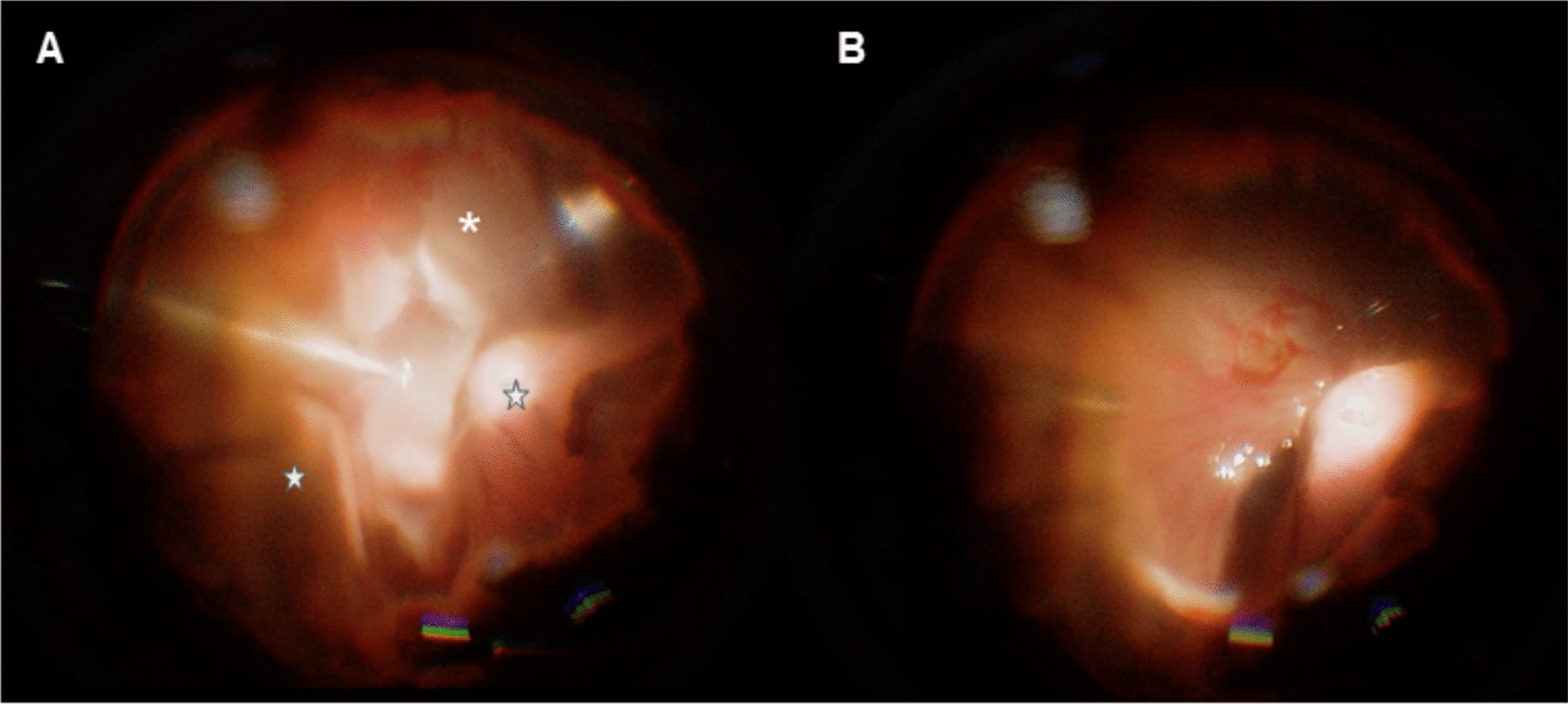
PFCL tamponade in a case of SCH. **A** Retinal incarceration (asterisk) and SCH (star) were observed during primary PPV. **B** Retina reattachment after PFCL tamponade

Traditional vitrectomy with primary silicone oil tamponade is often ineffective due to the following reasons: The presence of inflammatory cells, blood and proteins in the vitreous cavity can reduce the surface tension of silicone oil, leading to emulsification and diminished tamponade efficacy [28]. Additionally, choroidal attachment can be unachievable due to clot formation in the suprachoroidal space, leading to a significant reduction in the vitreous cavity (Fig. 3A). The irregular inner wall of the eyeball might result in inadequate silicone oil tamponade [29], and the absorption of SCH leads to oil underfilling, further reducing tamponade efficacy. PFCLs offer several advantages in these complex cases. Their high density provides continuous pressure on intraocular structures, aiding clot liquefaction and SCH absorption. Unlike silicone oil, PFCLs maintain retinal attachment even as SCH resorbs (Fig. 3B). Rizzo [29] demonstrated the use of PFCLs as a short-term tamponade in treating massive SCH, achieving a 100% retinal and choroidal reattachment rate with no complications during a 6-month postoperative follow-up.

PFCLs may offer significant benefits in managing choroidal injuries, particularly in cases of full-thickness choroidal damage. Choroidal rupture is often associated with hypotony and substantial intraocular hemorrhage from vortex vein tears, which are considered key risk factors for unfavorable outcomes, such as phthisis and enucleation [26], and increase the risk of OVAE [30]. Surgical reattachment of the choroid in eyes with OGIs and choroidal lacerations is particularly challenging due to the high collagen content and vasculature in the choroidal tissue, leading to tissue contraction and persistent bleeding. Furthermore, dense vitreous hemorrhage can obstruct proper evaluation of the peripheral retina, complicating fluid-air exchange. Re-bleeding after vitrectomy may occur, potentially accelerating silicone oil emulsification and the development of proliferative vitreoretinopathy (PVR), which raises the risk of recurrent retinal and choroidal detachment. PFCLs tamponade helps control bleeding through their gravitational properties and aids in the continuous displacement of hemorrhagic fluid, providing time for stabilization of intraocular structures. Additionally, due to their density and incompatibility with blood, PFCLs may support retinal and choroidal reattachment by separating blood, potentially reducing the risk of PVR formation. Furthermore, the surface tension of PFCLs help maintain their cohesiveness and might seal torn vortex veins, thereby mitigating the need for FAX.

PFCL tamponade has proven to be safe for clinical use. Several studies have used PFCLs as short-term postoperative tamponades (ranging from 2 to 15 days) and demonstrated their effectiveness in treating complex retinal detachments, PVR, melanoma resection, and massive SCH [29, 31–33]. Short-term PFCL tamponade has been proven safe by electrophysiological and morphological examinations [34, 35]. Sigler and Charles have demonstrated the safety and efficacy of mid-term perfluoro-n-octane (PFO) in treating recurrent inferior retinal detachments and retinal detachments complicated with PVR [36, 37]. Long-term retinal contact with perfluoro-octane has been well-tolerated, with only a macrophage response reported [38]. Viren et al. reported successful use of PFCL tamponade combined with keratoprosthesis in treating traumatic retinal detachment, leading to improved vision [39]. The duration of PFCL tamponade is not standardized and typically varies based on the surgeon’s preference. In our study, the tamponade duration was tailored based on the severity of ocular damage and the presence of SCH. A fixed tamponade duration of 7 or 14 days was used, in line with prior evidence suggesting maximal SCH liquefaction occurs within this time frame [40]. The decision to choice 7 or 14 days was based on the extent of ocular damage, with more severe cases, such as zone III globe rupture with extensive intraocular hemorrhage, significant SCH, choroidal rupture, typically receiving longer duration. All patients in our series achieved anatomical success with no signs of severe inflammation or toxicity. 

Another critical consideration is the prevention of OVAE, which can occur when pressurized air enters the systemic circulation via a torn vortex vein during fluid-air exchange, potentially leading to pulmonary air embolism. Symptoms typically manifest during or hours after routine vitrectomy procedures, including retinal detachment repair, melanoma resection or treatment of intraocular trauma. This can occur either due to slippage of the air infusion cannula into the suprachoroidal space or exposure of the vortex vein [4, 5, 41]. The risk of OVAE is heightened in severe OGIs due to extensive choroidal damage, making the vortex vein more accessible for air to pass during the FAX procedure. One way to bypass FAX is PFCL-oil exchange, which is commonly used to prevent retinal slippage in cases of retinal detachment with giant retinal tears. However, in our study, we chose not to perform PFCL-oil exchange for several reasons. First, in severe OGIs, extensive intraocular bleeding is almost always present, and fluctuations in intraocular pressure during the exchange process could exacerbate hemorrhage and obscure visualization, making it difficult to differentiate the PFCL-oil interface. Additionally, silicone oil’s low buoyancy is less effective than PFCLs in controlling hemorrhage, and blood can reduce the surface tension of silicone oil, leading to emulsification and diminished tamponade efficacy [28]. Second, over-pressurization of the vitreous cavity may occur due to the slow passive aspiration of PFCL, compared to the active injection of silicone oil, potentially increasing intraocular pressure [42]. Moreover, PFCL can interact with silicone oil, resulting in the formation of "sticky silicone oil," which may lead to photoreceptor toxicity if retained intraocularly [43].

Visual acuity improvement was limited in all cases, likely due to the extensive retinal and choroidal damage characteristic of severe OGIs. The cases presented reflect some of the poorest visual outcomes, as supported by prior prognostic studies, with many patients ultimately requiring enucleation [26]. This surgical approach aimed to preserve the eye through a safer and more manageable intervention. While anatomical success was achieved, long-term functional recovery in terms of visual acuity remains limited in severe OGIs.

The study has several limitations. First, the small sample size (n = 5) limits the generalizability of the results. Additionally, due to the highly heterogeneous nature of trauma cases, the outcomes observed in this study may not fully reflect the potential results for all patients with severe OGIs. Further research involving a larger, multi-center cohort with extended follow-up periods is necessary to validate the efficacy and safety of PFCL tamponade in a broader context. The follow-up periods for the patients vary and might be short for some cases. Longer follow-up would be necessary to assess the long-term outcomes. Furthermore, the study lacks a comparative element and is subject to biases inherent in its retrospective design. A prospective cohort study comparing different surgical approaches or tamponade agents would yield more robust and reliable outcomes.

In summary, short-term PFCL tamponade in a staged vitrectomy offers a viable strategy for managing severe OGIs. By stabilizing intraocular structures, preventing rebleeding, and reducing complications such as OVAE and PVR, this approach improves surgical feasibility and enhances anatomical outcomes. Additionally, it alleviates the profound psychological impact associated with enucleation. Further studies with larger cohorts and longer follow-up are needed to refine surgical protocols and assess long-term outcomes.

## Supplementary Information

Supplementary Material 1.

## Data Availability

No datasets were generated or analysed during the current study.

## Abbreviations

OGIs: Open globe injuries
PPV: Pars plana vitrectomy
SCH: Suprachoroidal hemorrhage
TCR: Traumatic choroidal rupture
OVAE: Ocular venous air embolism
FAX: Fluid-air exchange
PFCL: Perfluorocarbon liquids
IOP: Intraocular pressure
rTPA: Recombinant tissue plasminogen activator
OTS: Ocular trauma score
HM: Hand motion
LP: Light perception
NLP: No light perception
BCVA: Best-corrected visual acuity
PVR: Proliferative vitreoretinopathy
